# Abnormal IGF-Binding Protein Profile in the Bone Marrow of Multiple Myeloma Patients

**DOI:** 10.1371/journal.pone.0154256

**Published:** 2016-04-25

**Authors:** Liesbeth Bieghs, Malene Brohus, Ida B. Kristensen, Niels Abildgaard, Martin Bøgsted, Hans E. Johnsen, Cheryl A. Conover, Elke De Bruyne, Karin Vanderkerken, Michael T. Overgaard, Mette Nyegaard

**Affiliations:** 1 Department of Hematology, Aalborg Hospital, Aalborg University, Alborg, Denmark; 2 Department of Hematology and Immunology-Myeloma Center Brussel, Vrije Universiteit Brussel, Brussels, Belgium; 3 Department of Biomedicine, Aarhus University, Aarhus, Denmark; 4 Department of Chemistry and Bioscience, Aalborg University, Aalborg, Denmark; 5 Department of Haematology, Odense University Hospital, Odense, Denmark; 6 Division of Endocrinology, Metabolism and Nutrition, Endocrine Research Unit, Mayo Clinic, Rochester, MN, United States of America; University of Oxford, UNITED KINGDOM

## Abstract

Insulin-like growth factor (IGF) signalling plays a key role in homing, progression, and treatment resistance in multiple myeloma (MM). In the extracellular environment, the majority of IGF molecules are bound to one of six IGF-binding proteins (IGFBP1-6), leaving a minor fraction of total IGF free and accessible for receptor activation. In MM, high IGF-receptor type 1 expression levels correlate with a poor prognosis, but the status and role of IGF and IGFBPs in the pathobiology of MM is unknown. Here we measured total IGF1, IGF2, and intact IGFBP levels in blood and bone marrow samples from MM (n = 17), monoclonal gammopathy of undetermined significance (MGUS) (n = 37), and control individuals (n = 15), using ELISA (IGFs) and ^125^I-IGF1 Western Ligand Blotting (IGFBPs). MGUS and MM patients displayed a significant increase in intact IGFBP-2 (2.5–3.8 fold) and decrease in intact IGFBP-3 (0.6–0.5 fold) in the circulation compared to control individuals. Further, IGFBP-2 as well as total IGFBP levels were significantly lower in bone marrow compared to circulation in MM and MGUS only, whereas IGF1, IGF2, and IGFBP-3 were equally distributed between the two compartments. In conclusion, the profound change in IGFBP profile strongly suggests an increased IGF bioavailability in the bone marrow microenvironment in MGUS and MM, despite no change in growth factor concentration.

## Introduction

Multiple myeloma (MM) is an incurable plasma cell malignancy, characterized by the massive accumulation of terminally differentiated monoclonal plasma cells in the bone marrow (BM). MM cells are (in most cases) highly dependent on the BM microenvironment where growth and survival factors are secreted. During the last decade, the insulin-like growth factor (IGF) system has been demonstrated to play a prominent role within MM pathogenesis [1,2]. IGF1 has been shown to promote the growth, survival and migration of MM cells. High serum IGF1 levels and high IGF receptor type-1 (IGF-1R) expression have been linked to poor prognosis in MM patients, and osteoclasts have been reported to be a source of local IGF1 in MM [3–7]. Therapies targeting the IGF-1R have, however, failed to be translated into the clinic, most likely because of unspecific patient selection and lack of adequate biomarkers [8,9]. Recently, renewed interest in the IGF system has been generated by studies showing that IGF-1R inhibitors appear to be effective in overcoming drug resistance to known anti-myeloma agents, both *in vitro* and *in vivo* [10,11]. The IGF system consists of IGF1, IGF2, IGF-1R and six insulin-like growth factor binding proteins [IGFBP1-6]. Within the circulation, the majority of the IGF molecules are bound to IGFBPs leaving just a minor fraction free and bioavailable. Only free IGF binds to its receptor and initiates a signaling cascade resulting in proliferation and survival of cells. Therefore, the IGFBPs are major regulators of the IGF activity [12]. Until now, the level of IGFBPs in MM and the asymptomatic pre-malignant plasma cell disorder monoclonal gammopathy of undetermined significance (MGUS) [13], has been poorly described. Gene expression levels of the so-called IGFBP7 gene, also known as MAC25, Prostacyclin-Stimulating Factor, Tumor-Derived Adhesion Factor, or PGI2-Stimulating Factor, have recently been linked to poor prognosis [14]. IGFBP7 is not, however, a high affinity IGF binding protein and is not generally considered part of the extracellular IGF system [15].

The aim of this study was to systematically profile the extracellular components of the IGF system. This is the first investigation of the extracellular IGF components in paired samples of plasma from peripheral blood (PB) and BM in MM, MGUS and control individuals.

## Materials and Methods

The patients were diagnosed and peripheral blood (PB) and bone marrow (BM) plasma samples from patients and control individuals were collected at the Odense Hospital as previously described [16]. All MM patients were untreated newly diagnosed individuals. Patient characteristics are summarized in Table 1. All study subjects provided informed written consent. The study was approved by the Regional Medical Ethics Committee of the Odense University Hospital (protocol number S-20090093) and conducted according to the Helsinki declaration. Clinical characteristics are described by Kristensen et al. [16]. The gender distribution was comparable between the different groups.

**Table 1:** Patient characteristics.

	Number	Mean	Range
**Control individuals (n = 15)**			
Age (years)		63	50–76
Gender (M/F)	5/10		
**Monoclonal gammapathy of undetermined significance (n = 37)**			
Age (years)		73	46–88
Gender (M/F)	17/20		
**Multiple myeloma (n = 17)**			
Age (years)		72	52–86
Gender (M/F)	11/6		
**M-component type**			
IgG	11		
IgA	2		
**Light chain type**			
kappa	3		
lambda	0		
**NA**	1		
**ISS Stage**^**1**^			
Stage I	6		
Stage II	2		
Stage III	6		
**NA**	3		
**Adverse cytogenetics**			
13q	5		
t(4,14)	1		
17p	1		
**Plasma cells in BM (%)**		38,8	3–78

^1^ISS: International staging system, NA: not available

Total IGF1 and IGF2 levels were measured by ELISA, according to the manufacturer’s instructions (using the E20 & E30 kit, respectively, Mediagnost, Germany). Western Ligand Blotting (WLB) for determination of intact IGFBP levels was performed with ^125^I-IGF1 as previously described [17]. Briefly, protein from 1 μl blood or BM plasma samples were separated by sodium dodecyl sulfate-polyacrylamide gel electrophoresis (SDS-PAGE) using a 7.5–15% linear gradient under non-reducing conditions, and blotted onto a nitrocellulose membrane for 1.5 hours at 17 V. The membrane was incubated in 3% nonidet P-40/ tris-buffered saline (TBS) for 20–30 minutes, blocked (0.5% bovine serum albumin (BSA) in TBS) for 2 hours and labeled with ^125^I-IGF1 (R&D Systems Inc., Minneapolis, USA) overnight at 4°C. The membrane was exposed to a BioMax MR film (Kodak, Rochester, USA) for 1–2 weeks at -80°C by using a Dupont enhancing screen. IGFBP levels were quantified by densiomitry using the TotalLab Quant software (Isogen Life Sciences, The Netherlands). Each blot used for quantification included paired samples (PB and BM plasma) from at least two control individuals, and both MGUS and MM individuals. Western immunoblotting was performed as previously described [18], using a monoclonal mouse anti-IGFBP-2 antibody from Ansh Labs (TX, USA) as primary antibody and horseradish peroxidase-conjugated secondary goat anti-mouse antibody (the Jackson Laboratory). The blot was developed using enhanced chemiluminescence (Amersham Biosciences, NJ, USA). Graphical and statistical analyses for IGF1 and IGF2 levels were done using GraphPad Prism 5.0 software.

For analysis of the IGFBP levels, we assume that the level for each protein (IGFBP-2, IGFBP-3, and total IGFBP (sum of IGFBP-2 and IGFBP-3 band intensities)), can be modelled as

$${Protein}_{ijk}=e^{\left(\alpha_{ij}+B_{k}+A_{k│l}+\varepsilon_{ijk}\right)}$$

where α_*ij*_ represents the log-scale protein level for tissue *i* ϵ {BM, PB} and disease *j* ϵ {CON, MGUS, MM} and B_*k*_ denotes variation for *k*’th WLB (*k* ϵ {1,. . .. . .., 11}, A_*k│l*_ denotes individual person variation nested within each WLB for *l* ϵ {1,. . .. . .., *n*_*k*_}, and ε_*ijk*_ is the measurement errors. We assume the three variation terms are normal distributed with mean 0 and variance σ^2^_WLB_, σ^2^_P_, and σ^2^, respectively.

This means log Protein_*ijk*_ is a linear mixed model (LME). All statistical analyses of the model were performed with R version 3.2.0 (R Core Team. R: A language and environment for statistical computing, 2015. URL http://www.R-project.org/). In detail, the LME was estimated by the lme-function, from the R-package nlme. The model assumptions were controlled by quantile-quantile plots of the residuals. Standard deviations, confidence intervals, and P-values for contrasts on log-scale, e.g MGUS versus CON for PB,

$$\text{log}\;{Protein}_{PB,MGUS}-\;\text{log}\;{Protein}_{PB,CON}=\alpha_{PB,MGUS}-\alpha_{PB,CON}$$

were calculated by the glht-function in the R-package multcomp. Ratio between protein contrasts and their confidence intervals, e.g. MGUS versus CON for PB,

$$\frac{\text{log}\;{Protein}_{PB,MGUS}}{\text{log}\;{Protein}_{PB,CON}}\;=e^{\left(\alpha_{PB,MGUS}-\alpha_{PB,CON}\right)}$$

were calculated by the exponential function on appropriate contrasts and their standard deviation were calculated by the statistical delta-method. Confidence interval (CI) is given as the 95% CI. Non-parametric Spearman correlation analysis were performed using GraphPad Prism 5.0 software.

## Results

**Fig 1:**
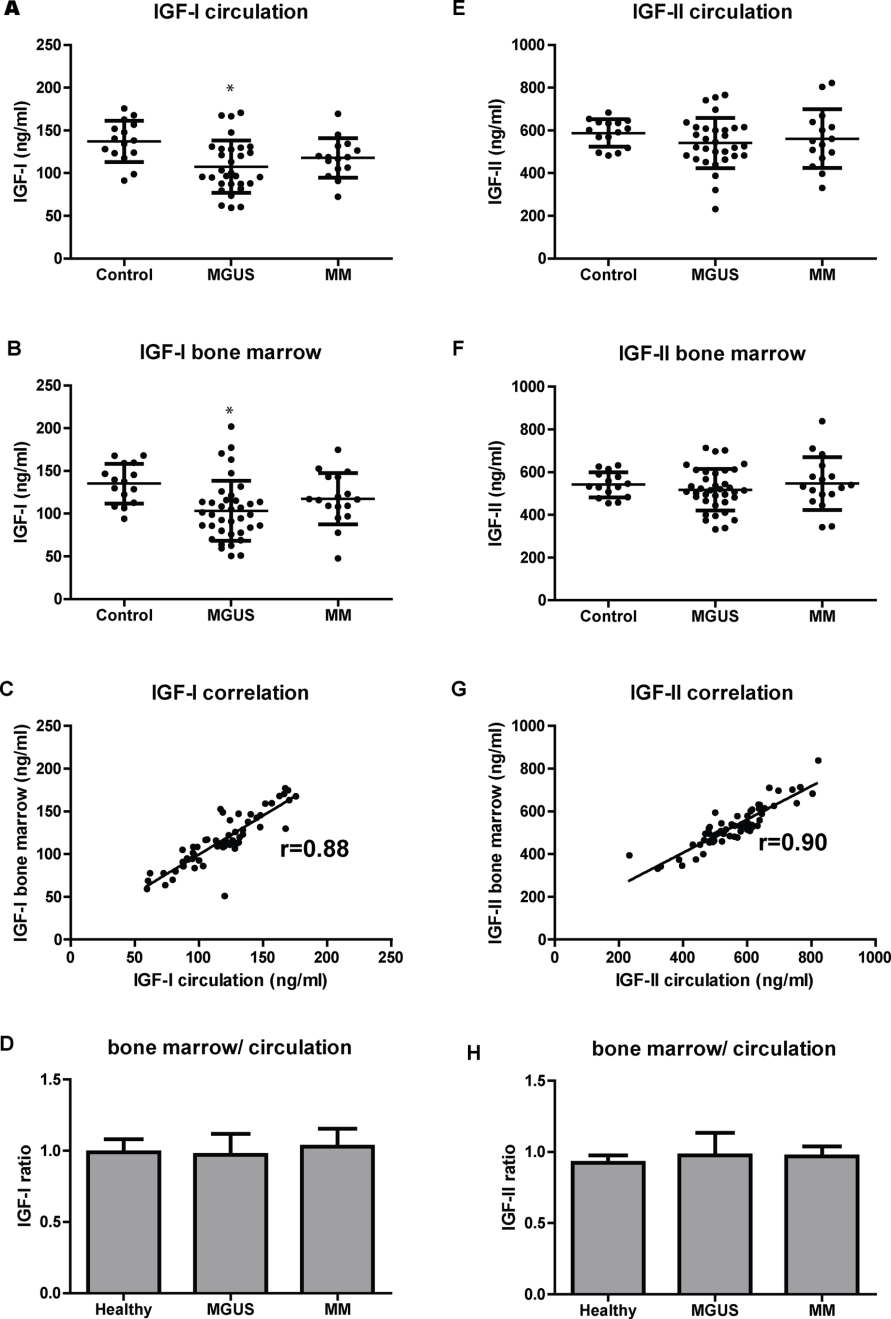
Total IGF1 and IGF2 levels in controls and patients with MGUS or MM. Levels of IGF1 measured by ELISA (A, B) and IGF2 (E, F) in plasma from circulation (A, E) and bone marrow (B, F). Correlation analysis of IGF1 (C) and IGF2 (G) between bone marrow and circulation. Mean pairwise ratio of bone marrow to circulating (for each individual) IGF1 (D) and IGF2 (H). Dots represent individual patients. Bars indicate standard deviations. (r) Pearson´s correlation coefficient. * p<0.01 compared to control.

First, total IGF1 and IGF2 levels were determined in the circulation and in the BM. Within the circulation, we found no significant difference in total IGFI levels between MM patients (117.7 ng/ml; CI 104.9–130.5) (n = 17) and control individuals (137.2 ng/ml; CI 123.8–150.6) (n = 15) (Fig 1A). The levels of total IGF1 in the BM were also comparable between MM patients (117.4 ng/ml; CI 102.9–132.9) and controls (135.0 ng/ml; CI 122.1–147.9) (Fig 1B). A small decrease in IGF1 level was observed in MGUS (n = 37) compared to controls, both for circulation (107.5 ng/ml; CI 96.1–119.0) and BM (103.2 ng/ml; CI 91.5–114.9) (p<0.01). For total IGF2, no difference was observed between MM, MGUS or control individuals, neither in the circulation, nor in the BM (Fig 1E and 1F). Moreover, we found that the average level of total IGF2 was four times higher than total IGF1. This is in accordance with previous findings [19]. The levels of total IGFs between the circulation and the BM were strongly correlated, independent of disease state (IGF1: r = 0.88, p<0.0001; IGF2: r = 0.90, p<0.0001) (Fig 1C and 1G). Further, IGF1 and IGF2 concentrations did not differ between the two compartments, suggesting that the total level of IGFs in the circulation can be used as a proxy for the levels in the BM (Fig 1D and 1H).

**Fig 2:**
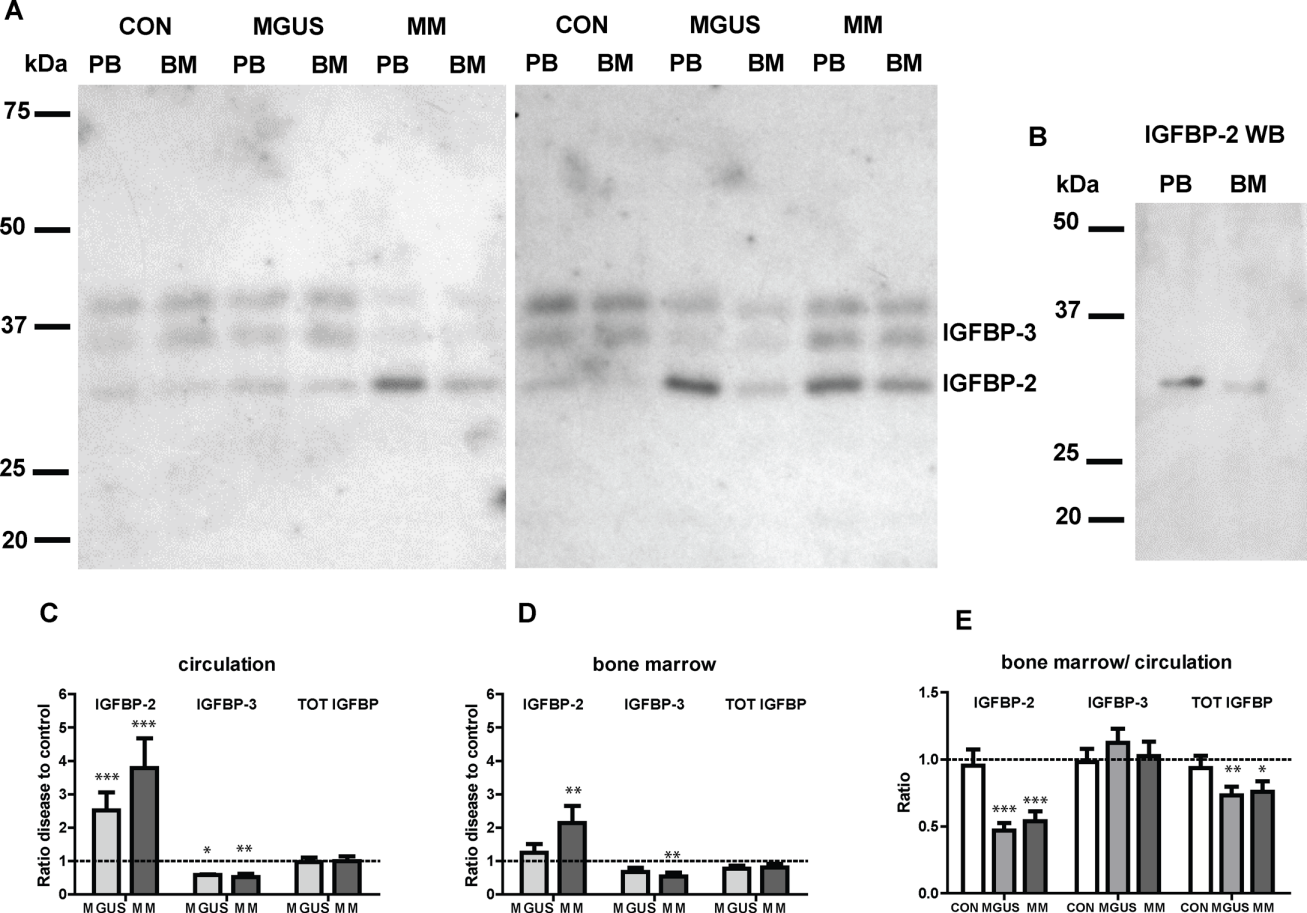
IGFBP levels and distribution in controls, MGUS and MM. A) Autoradiograph of two representative ^125^I-IGF Western ligand blots displaying plasma samples from the circulation (PB) and bone marrow (BM) taken from MGUS and MM patients and control (CON) individuals. The two top bands appearing at 38 and 42 kDa represent IGFBP-3. B) Western immunoblot analysis using a monoclonal IGFBP-2 primary antibody, confirming the identity of the 32-kDa band as IGFBP-2. C) The IGFBP disease to control ratio in the circulation. D) The IGFBP disease to control ratio in the bone marrow. E) Tissue distribution of the IGFBPs shown as the circulation to bone marrow ratio. * p<0.05, ** p<0.01, *** p<0.001

Next, we determined the presence and levels of intact IGFBPs, using ^125^I-IGF-1 western ligand blotting (WLB) on the same sample set. Fig 2A shows excerpts from two representative WLBs. We only observed 3 detectable IGFBPs bands, independent of disease state or compartment. The two high molecular weight IGFBP bands (MW 38 and 42 kDa) have previously been identified as different glycosylation forms of IGFBP-3 [20,21]. Western immunoblotting confirmed that the 32 kDa band was IGFBP-2 (Fig 2B). When analyzing the results from all WLBs, a different IGFBP profile was observed between control and disease states. In the circulation, a profound increase in the level of IGFBP-2 was observed in MGUS (2.5 fold; p<0.001) and MM (3.8 fold; p<0.001) compared to control samples (Fig 2C). In contrast, a decrease in IGFBP-3 levels was observed both for MGUS (down to 0.6 fold; p<0.05) and MM (down to 0.5 fold; p<0.01) compared to controls. Within the BM, the IGFBP-2 levels were significantly higher in MM patients only (2.1 fold; p<0.01) (Fig 2D), and IGFBP-3 levels were significantly lower in MM compared to control individuals (down to 0.5 fold; p<0.01). Total IGFBP levels did not show a statistically significant difference between control and MGUS or MM, neither in the circulation nor in the BM, although the total IGFBP levels in the BM tended to be lower (down to 0.8 fold for both MGUS and MM). The distribution of IGFBPs between the circulation and the BM compartment seemed to be fully equal in control individuals. However, we found that the ratio of IGFBP-2 between the BM and circulation was found significantly lower for MGUS and MM patients (down to 0.5 fold; p<0.001, for both), whereas no difference in the IGFBP-3 distribution was observed (Fig 2E). In addition, total IGFBP levels were significantly lower in the BM compartment for both MGUS (down to 0.7 fold; p<0.01) and MM (down to 0.8 fold; p<0.05) patients compared to the circulation. This strongly indicates that there is an increased IGF bioavailability in the BM microenvironment associated with the onset and progression of MM. In general, there was no significant correlation between plasma cell infiltration and the level of IGFBPs, except for the levels of circulating intact IGFBP-2, correlating positively with increased percentage of plasma cells (r = 0.38, p = 0.04) (S1 Fig).

## Discussion

**Fig 3:**
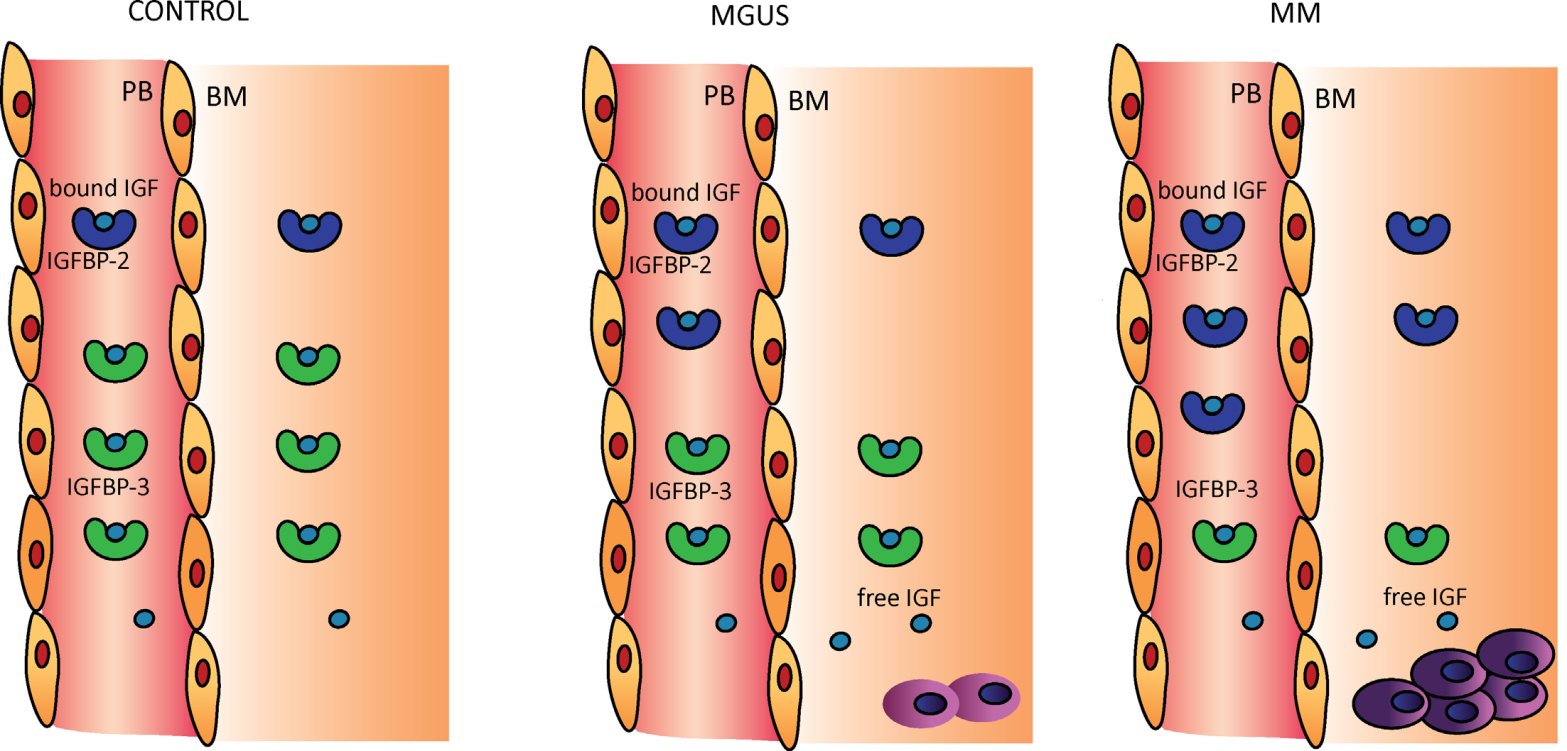
Schematic overview of IGF and IGFBP levels in controls, MGUS and MM patients. In Peripheral blood (PB): The level of IGFBP-2 significantly increases in MGUS and MM patients compared to controls, while IGFBP-3 decreases. The total IGFBP level is similar between MGUS, MM patients and control samples. In Bone marrow (BM): IGFBP-2 is significanlty increased in MM patients and IGFBP-3 is decreased in MGUS and MM patients. Total IGFBPs are lower in MGUS and MM patients. Tissue distribution: Total IGF1 and -2, IGFBP-2, IGFBP-3, and total IGFBP are distributed equally between the PB and BM in control individuals. In MGUS and MM patients, there are lower levels of IGFBP-2 and total IGFBP in the BM compared to PB. IGF1 and -2, and IGFBP-3 are equally distributed between compartments.

We are the first to describe the extracellular IGF system in the BM microenvironment in MGUS and MM patients. In this study, we demonstrate that in both MGUS and MM patients there is a large increase in circulating levels of intact IGFBP-2 concomitant with a marked decrease in IGFBP-3 levels. Interestingly, the increase in intact circulating IGFBP-2 correlated positively with the degree of plasma cell infiltration, suggesting that IGFBP-2 may be a novel marker for disease progression. Taken together with no to moderate changes in total IGF levels, there is a profound redistribution of IGFs from the major IGF carrier IGFBP-3 to IGFBP-2 in MGUS and MM. These changes are however, not reflected in the BM compartment, where the level of IGFBP-2 is significantly lower than in the circulation in both MGUS and MM, resulting in a lower total intact IGFBP level compared to the circulation (summarized in Fig 3). Within other types of cancer (e.g. prostate and breast cancer), the roles of IGFBP-3 and IGFBP-2 have been more thoroughly described, and demonstrated to convey both inhibitory and stimulating effects on tumor progression, depending on the tissue and type of cancer [12]. So far, nothing is known of their exact biological significance in MM. In non-MM bone, however, the IGFBPs are well established as key players for normal osteoblast and osteoclast function and bone turnover [22]. Bone formation is impaired in MM patients, and the abnormal IGFBP profile demonstrated here in the local BM microenvironment, could play a role in deregulation of bone remodeling in MM patients, since IGFBP-2 is known to be involved in bone formation and density determination [23]. We propose that the observed reduction in intact IGFBP-2 levels in the BM compared to the circulation, may be partially explained by localized IGFBP-2 proteolysis. Indeed metalloproteinases, such as pregnancy-associated plasma protein-A, have been demonstrated to be involved in this process [24]. In previous MM studies, increased expression of metalloproteinase genes were associated with poor prognosis [25]. Further research is needed to examine the possible link between metalloproteinases and our findings. Another possible explanation for the lower level of IGFBP-2 in the BM plasma, may be adhesion of the IGFBP-2 complex to the glycosaminoglycan-rich extracellular matrix [26]. Overall, the abnormal IGFBP profile identified in MGUS and MM suggest that the levels of bioavailable IGF is increased, in particular in the BM microenvironment in MM patients, despite unchanged or slightly lower total IGF concentrations (Fig 3). We propose that these perturbations in the extracellular IGF-system may be an important contributor to MM disease progression and treatment resistance. Further, our study suggests that targeting IGF signaling through modulation of the IGF binding protein profile, eg. using modified non-activatable IGFBPs, could represent a novel addition to therapeutic strategies for treating MM, as has been demonstrated for other cancer forms [27,28].

## Supporting Information

S1 Fig Correlation analysis of IGFBP levels and plasma cell infiltration. The degree of plasma cell infiltration was plotted against the level of IGFBP-2 (A, D), IGFBP-3 (B, E) and total IGFBP (C, F) in plasma from the circulation (A, B, C) and bone marrow (D, E, F). Non-parametric correlation analysis only identified a significant correlation for circulating IGFBP-2 levels and plasma cell infiltration (p<0.05). Dots represent individual patients. (r) Spearman’s correlation coefficient.
(PDF)
