# Supplementary material for: Abnormal IGF-Binding Protein Profile in the Bone Marrow of Multiple Myeloma Patients
Source: PLoS One. 2016 Apr 25;11(4):e0154256. doi: 10.1371/journal.pone.0154256 (PMC4844248; doi:10.1371/journal.pone.0154256)
Supplement: S1 Fig — The degree of plasma cell infiltration was plotted against the level of IGFBP-2 (A, D), IGFBP-3 (B, E) and total IGFBP (C, F) in plasma from the circulation (A, B, C) and bone marrow (D, E, F). Non-parametric correlation analysis only identified a significant correlation for circulating IGFBP-2 levels and plasma cell infiltration (p<0.05). Dots represent individual patients. (r) Spearman’s correlation coefficient. (PDF) [file pone.0154256.s001.pdf]

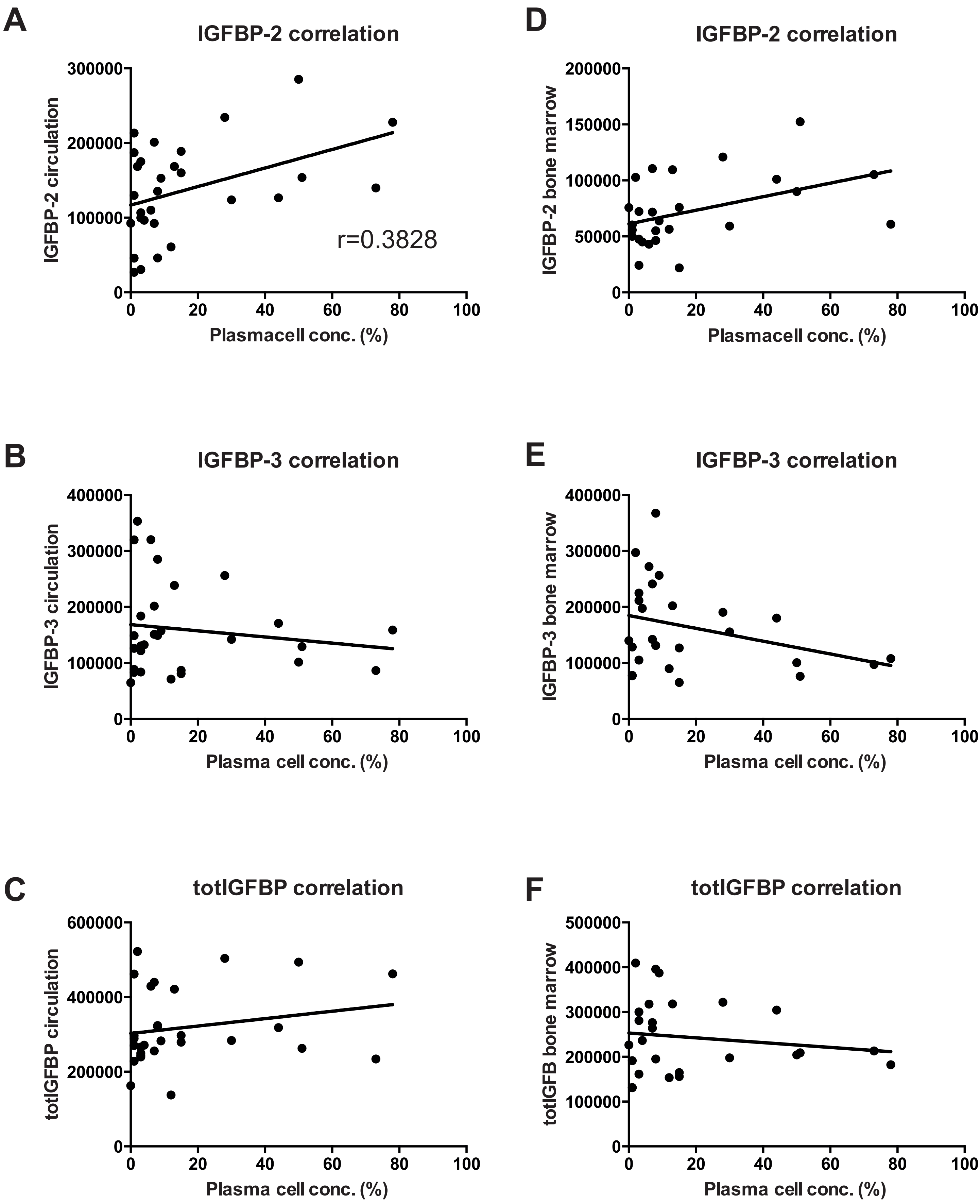

S1 Fig. Correlation analysis of IGFBP levels and plasma cell infiltration.
The degree of plasma cell infiltration was plotted against the level of IGFBP-2 (A, D), IGFBP-3 (B, E) and total IGFBP (C, F) in plasma from the circulation (A, B, C) and bone marrow (D, E, F). Non-parametric correlation analysis only identified a significant correlation for circulating IGFBP-2 levels and plasma cell infiltration ($p<0.05$). Dots represent individual patients. (r) Spearman's correlation coefficient.
